# MiR-302e attenuates allergic inflammation *in vitro* model by targeting RelA

**DOI:** 10.1042/BSR20180025

**Published:** 2018-05-28

**Authors:** Lifeng Xiao, Li Jiang, Qi Hu, Yuru Li

**Affiliations:** Department of Otolaryngology Head and Neck surgery, the First Affiliated Hospital of Harbin Medical University, Harbin 150001, People’s Republic of China

**Keywords:** allergic inflammation, cytokines, mast cells, microRNA-302e, RelA

## Abstract

Allergic inflammation is the foundation of allergic rhinitis and asthma. Although microRNAs are implicated in the pathogenesis of various diseases, information regarding the functional role of microRNAs in allergic diseases is limited. Herein, we reported that microRNA-302e (miR-302e) serves as an important regulator of allergic inflammation in human mast cell line, HMC-1 cells. Our results showed that miR-302e is the dominant member of miR-302 family expressed in HMC-1 cells. Moreover, the expression of miR-302e was significantly decreased in response to phorbol 12-myristate 13-acetate (PMA) and calcium ionophore A23187 or ovalbumin (OVA) stimulation. Overexpression of miR-302e blocked PMA/A23187 or OVA induced the increase in inflammatory cytokines levels, such as IL-1β, IL-6, tumor necrosis factor (TNF)-α and thymic stromal lymphopoietin, while miR-302 inhibition further promoted the release of these cytokines. Mechanistically, we found that miR-302e is a novel miRNA that targets RelA, a gene known to be involved in regulating inflammation, through binding to the 3′-UTR of RelA mRNA. Ectopic miR-302e remarkably suppressed the luciferase activity and expression of RelA, whereas down-regulation of miR-302e increased RelA luciferase activity and expression. Pharmacological inhibition of NF-κB reversed the augmented effect of miR-302e down-regulation on inflammatory cytokines level. Taken together, the present study demonstrates miR-302e limits allergic inflammation through inhibition of NF-κB activation, suggesting miR-302e may play an anti-inflammatory role in allergic diseases and function as a novel therapeutic target for the treatment of these diseases.

## Introduction

Allergic rhinitis (AR) is the most common noninfectious rhinitis all around the world [1]. AR patients suffer from nasal symptoms including loss of smell and taste, sneezing, lacrimation, and nasal obstruction and irritation [2]. This disease not only decreases the quality of life and routine work, but also negatively affects social economy [3]. Several important pharmacological agents have been used for AR treatment, such as leukotriene receptor antagonists, antihistamines, and intranasal steroids [2]. However, more than 20% AR patients do not show sufficient improvement [4]. Indeed, a population survey reported that up to 62% adults and 29% children undergo poor relief from pharmacotherapy [5]. Taking these data into account, AR is still a therapeutic challenge and more effective pharmacological agents are needed.

In fact, AR is a nasal inflammatory disease upon cutaneous inflammation driven by mast cells and mast cell-mediated Th2 cytokines [1,4]. Mast cells play a particularly important role in allergic diseases, including asthma and AR [6,7]. The percentage of mast cells activation is an indicator for the degree of allergic reaction in AR patients [8]. It has been shown that depletion of mast cells could interfere with chronic allergic inflammation of AR [9]. Activated mast cells secret inflammatory cytokines, such IL-1β, IL-6, tumor necrosis factor (TNF)-α, and thymic stromal lymphopoietin (TSLP) via NF-κB signaling cascades, which contribute to the influx of immune cells and subsequently accelerate inflammatory reactions [7,10]. Thus, these findings together implicate that prevention of mast cell activation and its cytokines production is an effective approach for AR treatment.

MicroRNAs (miRNAs) are a class of endogenous, small (22 nucleotides in length) and noncoding RNAs that post-transcriptionally regulate the target genes expressions by binding to the 3′-untranslated region (3′-UTR) [11]. Clinical trials and animal experiments demonstrate that miRNAs may be a promising biomarker for AR [11–13]. Suojalehto et al. [12] found that miR-498, miR-187, miR-143, miR-874, and miR-886-3p levels were increased, while let-7e, miR-18a, miR-126, miR-155 and miR-224 were decreased in the asthmatic patients. These alterations of miRNAs were associated with the severity of asthma. Our previous *in vivo* study showed that MiR-133b could alleviate allergic symptom of AR mice by inhibition of Nlrp3 inflammasome-meditated inflammation [14]. Further, recent study also found that miR-122–SOCS1–JAK2 axis was involved in the regulation of allergic inflammation [15]. These findings suggest that miRNAs could functionally modulate allergic inflammation [10,14,15]. In the present study, we found that miR-302e is the dominant member of miR-302 family expressed in HMC-1 cells, and it was decreased after allergen stimulation, which promotes us to speculate that miR-302e may play a critical role in allergic inflammation. MiR-302e belongs to the miR-302 family, which consists of five members, miR-302a, miR-302b, miR-302c, miR-302d, and miR-302e. The biological functions of this miRNA family were mainly focused in stem cells [16,17]. MiR-302 is highly expressed in human embryonic stem cells (hESCs), and overexpression of miR-302 cluster can promote somatic cell reprograming and maintain hESCs stemness and self-renewal [16,18,19]. However, there are few studies addressing the effect of miR-302 on allergic inflammation. Our present study indicates that miR-302e may be a novel therapeutic target for the treatment of allergic inflammation and AR.

## Methods and materials

### Materials and reagents

Iscove’s modified Eagle’s medium (IMDM), fetal bovine serum (FBS), streptomycin, penicillin, OptiMEM I medium, and Lipofectamine 2000 were purchased from Invitrogen (CA, U.S.A.). Dimethyl sulfoxide (DMSO), phorbol-12-myristate-13-acetate (PMA), calcium ionophore A23187, ovalbumin (OVA), dexamethasone (Dex), and BAY11 were obtained from Sigma Chemical Co. (MO, U.S.A.). RIPA lysis buffer, Enhanced BCA Protein Determination Kit, and anti-rabbit FITC antibody were from Beyotime (Shanghai, China).

### Cell culture

The human mast cell lines, HMC-1 cells, were a generous kit from Zhiliang Yu (Second Military Medical University, China) and cultured in IMDM containing 10% FBS, 100 mg/ml streptomycin, and 100 units/ml penicillin at 37°C in 5% CO_2_ atmosphere at 95% relative humidity.

### Quantitative real-time PCR

Total RNA from HMC-1 cells were isolated using Trizol reagent (Thermo Fisher Scientific, Yokohama, Japan) according to the manufacturer’s protocols. Two micrograms of RNA was reverse-transcribed using a RevertAid First Strand cDNA Synthesis Kit (Thermo Fisher Scientific). Real-time PCR was performed using SYBR Green PCR Master Kit (Bio-Rad Laboratories, CA, U.S.A.) on a LightCycler 480 qPCR System (Roche, Basel, Switzerland). To measure miR-302a, miR-302b, miR-302c, miR-302d, and miR-302e expression, cDNA was amplified using miRcute miRNA qPCR kit (TIANGEN, Beijing, China). The specific target gene primers were purchased from RiboBio Co., Ltd. (Guangzhou, China). The real-time PCR experiment was executed in triplicate and the relative mRNA expression index was normalized with GAPDH or U6.

### MiRNA transfection

MiR-302e mimics or miR-302e inhibitor or their corresponding negative controls (RiboBio Co., Ltd.) were diluted with OptiMEM I medium and then transfected into HMC-1 cells with Lipofectamine 2000 in accordance to supplier’s protocols. After 48 h transfection, cells were treated with PMA (20 nM) plus A23187 (1 μM) or OVA (5 mg/ml) for another 24 h. The concentration of treatment was selected according to a previous report [20].

### ELISA

To evaluate the inflammatory response, cytokines (IL-1β, IL-6, TNF-α, and TSLP) were assayed in cell culture medium using ELISA kits (Abcam, MA, U.S.A.). Procedures were performed according to the manufacturer’s instructions.

### Luciferase reporter assay

Luciferase reporter assay was performed to predict the direct binding of miR-302e to the target gene RelA. The human RelA 3′-UTR containing miR-302e binding site was generated from HMC-1 genomic DNA and then cloned into the pMIR vector (RiboBio Co., Ltd.), referred to as wild-type RelA 3′-UTR. The mutant 3′-UTR of the RelA gene by substitution of 6 bp from seed region of miR-302e was directly synthesized and inserted into the equivalent reporter vector. HMC-1 cells (2 × 10^5^/well) were seeded in 24-well plates and co-transfected with 3′-UTR of RelA (with either wild-type or mutant luciferase vector) and miR-302e mimics or miR-302e inhibitor using Lipofectamine 2000. Forty-eight hours later, the cells were collected and the luciferase activity was assessed using Dual-Luciferase Reporter Assay System (Promega).

### Western blotting

HMC-1 cells were lysed with RIPA lysis buffer containing protease and phosphatase inhibitors (Roche Applied Science, IN, U.S.A.) at 4°C. Cytoplasmic and nuclear proteins of aortas were extracted using a Nuclear/Cytosol Fractionation Kit (BioVision, CA, U.S.A.) according to the manufacturer’s protocols. The protein concentration was determined by Enhanced BCA Protein Assay Kit. Equal amount of protein (50 μg) was heated at 95°C for 5 min and separated by 10% sodium dodecyl sulfate/polyacrylamide gel electrophoresis (SDS/PAGE) gels. Protein was then transferred onto polyvinylidene fluoride (PVDF) membranes (Millipore, MA, U.S.A.), which was subsequently blocked by 5% nonfat milk for 1 h. Afterward, the membranes were incubated with the following primary antibodies: RelA (1:500), GAPDH, and Lamin B (1:1000) (Cell Signaling Technology, MA, U.S.A.). The immunoreactive proteins were detected with horseradish peroxidase-conjugated secondary antibodies (Santa Cruz Biotechnology, CA, U.S.A.) and an enhanced chemiluminescence reagent (Pierce Biotech, IL, U.S.A.). Densitometry of bands was quantified using ImageJ software (NIH, Maryland, U.S.A.).

### Immunofluorescent staining

To detect RelA nuclear translocation in HMC-1 cells, cells were fixed and labeled with rabbit-anti-RelA (1:100) antibody. After incubation with the primary antibody overnight at 4°C, cells were then washed three times with PBS and incubated with anti-rabbit FITC antibody for labeling RelA (1:200) for 1 h at room temperature. Fluorescent images were acquired using the Zeiss Axioplan2 fluorescence microscope (Munich, Germany).

### Statistical analysis

All data were presented as mean value ± standard error of mean (SEM) and compared by two-tailed Student’s *t* test or one-way ANOVA, followed by the Bonferroni multiple comparison test. Statistical analysis was performed by SPSS 18.0 software (SPSS Inc., IL, U.S.A.). *P*<0.05 was considered statistically significant.

## Results

### Decreased expression of miR-302e in allergen-activated HMC-1 cells

**Figure 1:**
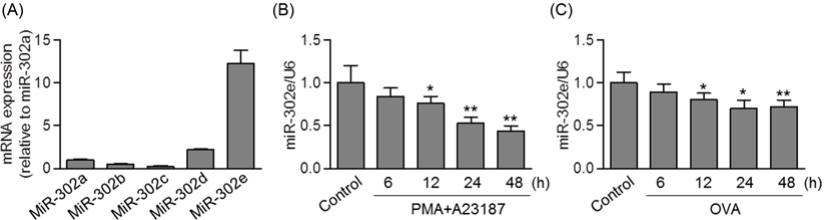
Decreased expression of miR-302e in mast cells after PMA/A23187 treatment (**A**) Quantitative real-time PCR analysis of miR-302a, miR-302b, miR-302c, miR-302d, and miR-302e levels in human mast cell line, HMC-1 cells. (**B** and **C**) The cells were treated with PMA (20 nM) plus A23187 (1 μM) or ovalbumin (OVA, 5 mg/ml) for 6, 12, 24, or 48 h. The expression of miR-302e was determined by quantitative real-time PCR; *P<0.05, **P<0.01 vs. control, *n*=6.

MiR-302 family comprises five miRNAs: miR-302a, miR-302b, miR-302c, miR-302d, and miR-302e. To examine the expression pattern of miR-302 family in HMC-1 cells, quantitative real-time PCR was performed. The results showed that the expression of miR-302e was approximately 8-fold higher than that of miR-302a and 5-fold higher than that of miR-302d. The expression of miR-302b and miR-302c was too faint to be detected (Figure 1A). These data suggest that miR-302e is the dominant member of miR-302 family expressed in mast cells. Given that mast cells play critical roles in the pathogenesis of AR and PMA/A23187 can potently cause allergic responses by mast cells [21], we initially explored the role of miR-302e in PMA/A23187-activated HMC-1 cells *in vitro*. PMA/A23187 induced miR-302e down-regulation in HMC-1 cells by a time-dependent manner (Figure 1B). Moreover, we also stimulated HMC-1 cells with 5 mg/ml of OVA for further analysis. Similarly, OVA treatment led a time-dependent decrease in miR-302e level (Figure 1C). In addition, no compensatory expression of miR-302a, miR-302b, miR-302c, and miR-302d was observed upon allergen stimulation (Supplementary Figure S1). These data indicate that miR-302e may play a functional role in the regulation of allergic responses.

### Overexpression of miR-302e blocked allergen-induced allergic inflammation

**Figure 2:**
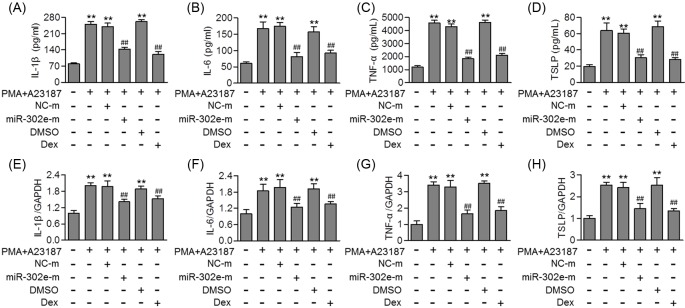
MiR-302e overexpression inhibited allergen-induced allergic inflammation in mast cells (**A**–**D**) HMC-1 cells were pretreated with miR-302e mimics (miR-302e-m, 40 nM) or mimics negative control (NC-m) for 48 h, or dexamethasone (Dex, 100 nM) for 1 h before PMA (20 nM) plus A23187 (1 μM) stimulation for further 24 h. ELISA analysis of the level of IL-1β (A), IL-6 (B), TNF-α (C), and TSLP (D). (**E**–**H**) The mRNA level of IL-1β (E), IL-6 (F), TNF-α (G), and TSLP (H) was examined by quantitative real-time PCR; **P<0.01 vs. control; ^##^*P*<0.01 vs. PMA + A23187, *n*=5.

To investigate whether allergen-decreased miR-302e expression is essential for allergic inflammation, HMC-1 cells were transfected with miR-302e mimics or mimics negative control before PMA/A23187 or OVA stimulation. Quantitative real-time PCR confirmed that miR-302e was successfully overexpressed in HMC-1 after miR-302e mimics transfection without affecting miR-302a, miR-302b, miR-302c, and miR-302d mRNA levels (Supplementary Figure S2A). As shown in Figure 2A–D, PMA/A23187 significantly increased the level of inflammatory cytokines, such as IL-1β, IL-6, TNF-α, and TSLP. However, up-regulation of miR-302e markedly inhibited the increase in these cytokines. Consistently, miR-302e overexpression also suppressed the mRNA level of IL-1β, IL-6, TNF-α, and TSLP induced by PMA/A23187 (Figure 2E–H). As expected, Dex, which was used as a positive control, exerted similar inhibitory effect on inflammatory cytokines level (Figure 2). In addition, up-regulation of miR-302e was also associated with reduced IL-1β, IL-6, TNF-α, and TSLP mRNA level upon OVA treatment (Supplementary Figure S3A–D).

### Inhibition of miR-302e promoted allergic inflammation in HMC-1 cells

**Figure 3:**
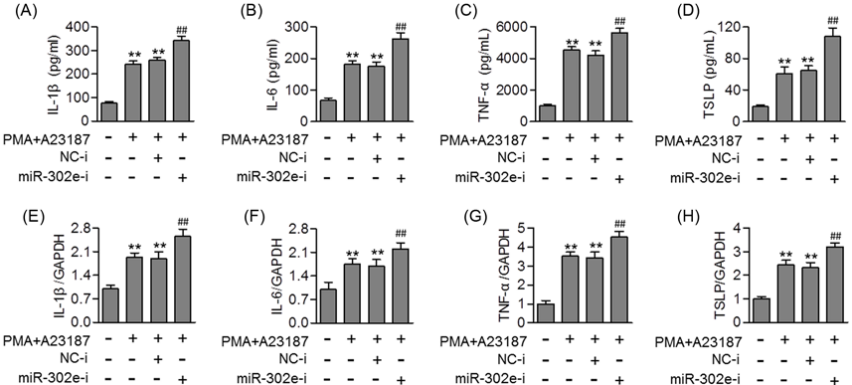
MiR-302e inhibition enhanced allergic inflammation induced by PMA/A23187 (**A**–**D**) HMC-1 cells were transfected with miR-302e inhibitor (miR-302e-i, 40 nM) or inhibitor negative control (NC-i) for 48 h and then treated with PMA (20 nM) plus A23187 (1 μM) for 24 h. The level of IL-1β (A), IL-6 (B), TNF-α (C), and TSLP (D) was determined by ELISA assay. (**E**–**H**) Quantitative real-time PCR analysis of the mRNA level of IL-1β (E), IL-6 (F), TNF-α (G), and TSLP (H); **P<0.01 vs. control; ^##^*P*<0.01 vs. PMA + A23187, *n*=5.

To further confirm the effect of miR-302e on allergic responses, we inhibited miR-302e expression in HMC-1 prior to allergen treatment. MiR-302e inhibitor transfection significantly decreased miR-302e expression, but had no effect on miR-302a, miR-302b, miR-302c, and miR-302d expression levels (Supplementary Figure S2B). The results of ELISA showed that the PMA/A23187 induced the release of IL-1β, IL-6 and TNF-α, and TSLP was markedly enhanced after miR-302e inhibition (Figure 3A–D). Moreover, knockdown of miR-302e also further increased the mRNA level of IL-1β, IL-6, TNF-α, and TSLP induced by PMA/A23187 or OVA (Figure 3E–H and Supplementary Figure S3E–H). Overall, the results demonstrate that miR-302e expression may be critical for allergic inflammation.

### MiR-302e targeted at RelA 3′-UTR

**Figure 4:**
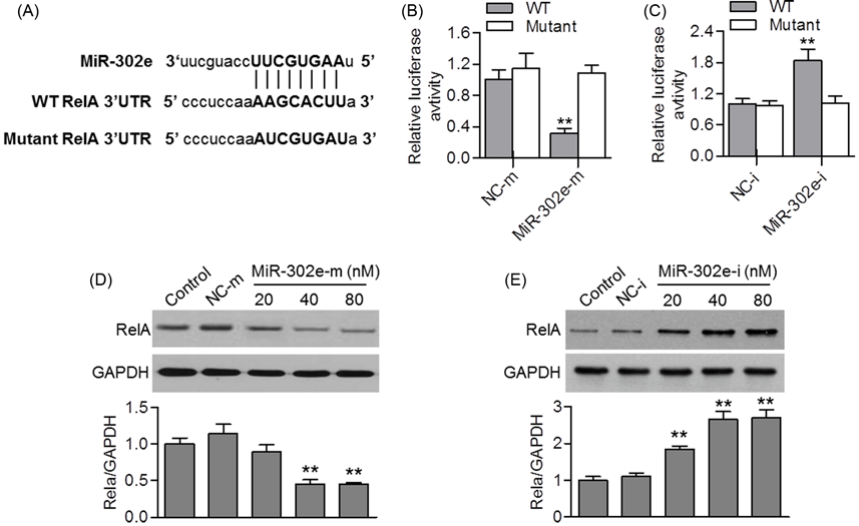
MiR-302e negatively regulates RelA expression (**A**) Predicted miR-302e seed matches to the sequence in the 3′-UTR of RelA. The complementary sequences were shown. (**B** and **C**) HMC-1 cells were co-transfected with luciferase reporter containing RelA 3′-UTR or the mutant one, and miR-302e mimics (B) or miR-302e inhibitor (C). Dual luciferase activity assay was performed to test the luciferase activity of RelA 3′-UTR; **P<0.01 vs. NC-m, *n*=5. (**D** and **E**) Western blotting analysis of RelA protein expression in HMC-1 cells transfected with miR-302e mimics (D) and miR-302e inhibitor (E) for 48 h; **P<0.01 vs. control, *n*=4.

To look for the target gene of miR-302e in HMC-1 cells, we used the miRNA prediction software (TargetScan 7.0, www.Targetscan.org) to search the potential target genes. The results showed that RelA, a gene known to be involved in regulating inflammation, was the potential target gene of miR-302e. As displayed in Figure 4A, an 8 bp fragment of RelA 3′-UTR is complementary to the miR-302e seed sequence. By co-transfection with miR-302e mimics, miR-302e inhibitor or their corresponding negative controls, and RelA 3′-UTR or the mutant one in HMC-1 cells, the luciferase assay showed that up-regulation of miR-302e significantly decreased the luciferase activity of RelA 3′-UTR. However, miR-302e mimics failed to affect the luciferase activity elicited by the reporter carrying the RelA 3′-UTR with the mutant miR-302e-binding site (Figure 4B). Additionally, miR-302e inhibition significantly increased the luciferase activity of RelA 3′-UTR, but this enhanced effect was also abrogated in cells transfected with mutated RelA 3′-UTR (Figure 4C). Furthermore, the effect of miR-302e on endogenous RelA protein expression was determined. Consistently, up-regulation of miR-302e reduced RelA protein expression, whereas miR-302e inhibition increased RelA expression (Figure 4D,E). These data indicate that miR-302e negatively regulates RelA expression.

### MiR-302e inhibited activation of NF-κB in HMC-1 cells

**Figure 5:**
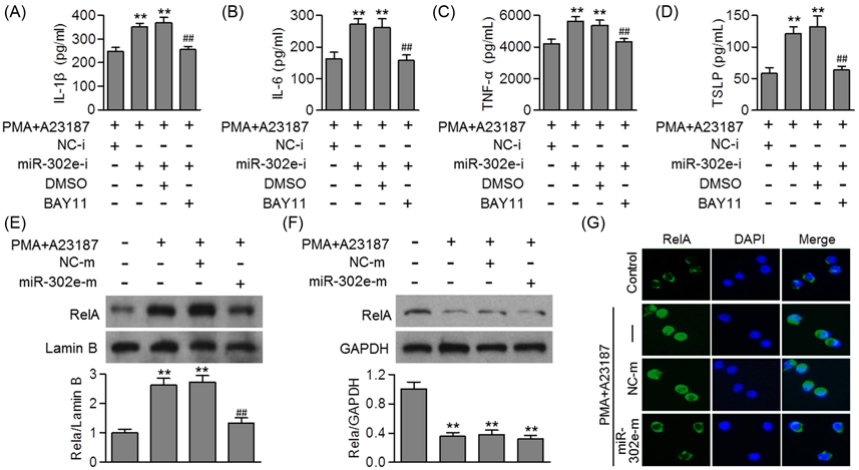
MiR-302e suppressed NF-κB activation (**A–D**) HMC-1 cells were pretreated with miR-302e inhibitor or inhibitor negative control for 48 h, or with BAY11 (20 μM) for 1 h, prior to PMA (20 nM) plus A23187 (1 μM) stimulation. The level of IL-1β (A), IL-6 (B), TNF-α (C), and TSLP (D) was determined by ELISA; **P<0.01 vs. PMA+A23187; ^##^*P*<0.01 vs. PMA + A23187 + miR-302e-i, *n*=4. (**E** and **F**) The cells were transfected with miR-302e mimics or mimics negative control for 48 h and then treated with PMA plus A23187 for 24 h. Nuclear (E) and cytosol (F) proteins were isolated and detected by Western blotting using RelA antibody. (**G**) Representative images of RelA distribution in HMC-1 cells; **P<0.01 vs. control; ^##^*P*<0.01 vs. PMA + A23187, *n*=6.

To confirm that whether RelA is the direct target of miR-302e that mediates allergic inflammation, we employed a pharmacological inhibitor of NF-κB, and then measured its effects on the release of inflammatory cytokines. The enhanced effects of miR-302e inhibition on inflammatory cytokines release were completely inhibited by NF-κB inhibitor, BAY11 (Figure 5A–D), further suggesting the critical role of RelA in miR-302e-attenuated allergic inflammation. We next investigated the effect of miR-302e on NF-κB activation, which is important for regulating inflammatory cytokines production by activated mast cells during allergic inflammation [22–24]. Western blotting results showed that the translocation of RelA from cytoplasm to nucleus was increased after PMA/A23187 treatment for 24 h, indicating the activation of NF-κB. Following miR-302e overexpression, the increased level of RelA in nucleus was inhibited, but the level of RelA in cytoplasm remained the same (Figure 5E,F). This was further confirmed by immunofluorescent staining using anti-RelA antibody (Figure 5G). These data indicate that miR-302e ameliorates allergic inflammation at least in part through inhibition of NF-κB signaling pathway.

## Discussion

Accumulating evidences have suggested that miRNAs exert robust effects on immune response by functionally targeting multiple target genes in a wide variety of cells [13,25]. Considering the higher stability of miRNA than other mRNA moieties or even some proteins, this stability is yet to be exploited for therapeutic approach for allergic diseases [14]. For example, miR-21 knockout mice showed reduced allergic inflammation in lung after allergen stimulation [26], while miR-155 knockout mice revealed increased passive cutaneous anaphylaxis responses in mast cells [27]. Moreover, it is worthy to note that the aberrant level of miRNAs may implicate important clinicopathological significances of AR [12]. MiR-106b in dendritic cells and miR-133a in bronchial smooth muscle cells were down-regulated after OVA challenge, while miR-221 and miE-485 were shown to increase in the lung tissues of an OVA-induced murine asthma model [28–30]. These changes of miRNAs play a key role in allergic inflammation. Additionally, miR-125b was found to increase in the mucosal epithelium of chronic rhinosinusitis patients, and this overexpression increased type I IFN release by negatively regulating 4E-BP-1 protein expression [31]. In monocytes isolated from 6-year-old children with AR, miR-21 was down-regulated, which reduced the severity of the inflammatory response [26,32]. Moreover, our recent study observed that miR-133b expression was reduced in OVA-induced AR mice, while restoration of this miRNA could attenuate allergic inflammation by targeting Nlrp3 [14]. Herein, we provide *in vitro* evidence that miR-302e is the dominant miR-302 family member expressed in HMC-1 cells and dramatically regulated in response to PMA/A23187 or OVA treatment. In support, using complementary gain- and loss-of-function approaches, we demonstrate that miR-302e inhibits allergic inflammation by targeting RelA. In light of these findings, our study suggests that miR-302e may be a novel regulator of RelA and allergic inflammation.

Mast cells play a foundational role in the acute phase of AR [7,8]. Activation of mast cells leads to allergic inflammatory response and increases the infiltration of immune cells, such as neutrophils, dendritic cells, and eosinophils [4,6,7]. Thus, mast cell degranulation has been suggested to the major target of antiallergic drugs. Several miRNAs have been reported to regulate cellular function of mast cells. For example, overexpression of miR-375 in esophageal epithelium inhibited mast cell activation [33]. Mayoral et al. [34] demonstrated that miR-221/222 was up-regulated in activated mast cells and inhibited cell cycle and cytoskeleton. Additionally, the degranulation of mast cells was also attenuated by miR-155 and miR-223 [27,35]. In our study, mast cell activation was induced *in vitro* by PMA and A23187, which are protein kinase C activator and calcium ionophore respectively. It has been documented that the level of protein kinase C family and intracellular calcium is important for allergen-mediated mast cell activation [4,20]. Upon PMA/A23187 challenge, mast cells can produce inflammatory cytokines (TNF-α, TSLP, IL-1β, IL-5, IL-6, IL-8, and IL-13) via NF-κB pathway [22,36]. Indeed, our results showed that PMA/A23187 treatment resulted in a dramatic increase in IL-1β, IL-6, TNF-α, and TSLP levels, which was in agreement with previous studies [4,20]. Importantly, miR-302e overexpression led to a marked inhibition of IL-1β, IL-6, TNF-α, and TSLP levels in PMA/A23187-treated HMC-1 cells. However, down-regulation of miR-302e promoted the release of the above cytokines. Furthermore, we also treated HMC-1 cells with OVA. Expectedly, elevation of miR-302e inhibited, whereas knockdown of miR-302e potentiated OVA-induced the increase in IL-1β, IL-6, TNF-α, and TSLP mRNA levels. Collectively, these data demonstrate the inhibitory effect of miR-302e on allergic inflammation.

To determine the mechanisms by which allow miR-302e to inhibit allergic inflammation in HMC-1 cells, custom prediction of TargetScan was used to predict the targets of miR-302e. The potential targets of miR-302e that could be involved in inflammation led us to focus on RelA, also known as p65, a member of the NF-κB family, which regulates many inflammatory cytokines and reactions [37]. Inhibition of NF-κB transcriptional activity in mast cell nucleus can significantly suppress the expression of adhesion molecules and inflammatory cytokines [24]. Several miRNAs have been evidenced to control RelA through binding to specific target sequences in the 3′-UTR region, as shown for miR-124, miR-221, and miR-186 [38–40]. In the present study, we found an inverse regulation of RelA by miR-302e. Ectopic miR-302e remarkably suppressed RelA expression and luciferase activity, whereas inhibition of miR-302e was associated with opposite results. This demonstrates that miR-302e directly targets to the 3′-UTR of RelA. Simultaneously, the enhanced level of inflammatory cytokines in PMA/A23187-treated HMC-1 cells with miR-302e down-regulation has been similarly reversed by pharmacological inhibition of NF-κB. Therefore, it strongly indicates that miR-302 inhibits PMA/A23187-induced allergic inflammation by targeting RelA. Interestingly, although miR-302e overexpression inhibited RelA translocation and reduced the abundance of RelA in nucleus, the cytoplasmic level remained unchanged. These results suggest that the inhibitory effect of miR-302e on inflammatory cytokines may be partially due to its ability to suppress NF-κB transcriptional activity in mast cell nucleus.

In summary, the present study demonstrates that miR-302e is down-regulated in HMC-1 cells in response to PMA/A23187. MiR-302e overexpression limits allergic inflammation through inhibition of NF-κB activation. These results reveal a novel anti-inflammatory role of miR-302e in activated mast cells, suggesting that miR-302e may be a promising therapeutic target for the treatment of allergic diseases, such AR.

## Supporting information

**Figure S1.** Effects of PMA/A23187 or OVA treatment on miR-302a, miR-302b, miR-302c and miR-302d level. (A-H) HMC-1 cells were treated with PMA (20 nM) plus A23187 (1 μM) or ovalbumin (OVA, 5 mg/mL) for 6, 12, 24 or 48 h. The expression of miR-302a (A and E), miR-302b (B and F), miR-302c (C and G) and miR-302d (D and H) was determined by quantitative real-time PCR.

**Figure S2.** Effect of MiR-302e mimics or inhibitor on the expression of miR-302 family members. (A and B) HMC-1 cells transfected with miR-302 mimics (miR-302e-m, 40 nM) (A) or miR-302 inhibitor (miR-302e-i, 40 nM) (B) for 48 h. The expression of miR-302a, miR-302b, miR-302c, miR-302d and miR-302e was determined by quantitative real-time PCR. **P<0.01 vs. NC-m or NC-i, n=6.

**Figure S3.** Effects of miR-302e on OVA-induced the mRNA level of cytokines. (A) HMC-1 cells were transfected with miR-302e mimics (miR-302e-m, 40 nM), miR-302e inhibitor (miR-302e-i, 40 nM), mimics negative control (NC-m) or inhibitor negative control (NC-i) for 48 h and then treated with ovalbumin (OVA, 5 mg/mL) for 24 h. The mRNA level of IL-1β (A and E), IL-6 (B and F), TNF-α (C and G) and TSLP (D and H) was examined by quantitative real-time PCR. **P<0.01 vs. control; ##P<0.01 vs. OVA, n=6.

## Abbreviations

AR: allergic rhinitis
GAPDH: glyceraldehyde-phosphate dehydrogenase
hESC: human embryonic stem cell
HMC: human mast cells
IL: interleukin
IMDM: Iscove’s modified Eagle’s medium
NF-kB: nuclear factor-kB
OVA: ovalbumin
PMA: phorbol-12-myristate-13-acetate
TNF: tumor necrosis factor

## References

[1] Rosati M.G. and Peters A.T. (2016) Relationships among allergic rhinitis, asthma, and chronic rhinosinusitis. Am. J. Rhinol. Allergy 30, 44–47 10.2500/ajra.2016.30.4252 26867529 PMC5517779

[2] Bousquet J., Khaltaev N., Cruz A.A., Denburg J., Fokkens W.J., Togias A. (2008) Allergic rhinitis and its impact on asthma (ARIA) 2008 update (in collaboration with the World Health Organization, GA(2)LEN and AllerGen). Allergy 63, 8–160 10.1111/j.1398-9995.2007.01620.x 18331513

[3] Valovirta E., Myrseth S.E. and Palkonen S. (2008) The voice of the patients: allergic rhinitis is not a trivial disease. Curr. Opin. Allergy Clin. Immunol. 8, 1–9 10.1097/ACI.0b013e3282f3f42f 18188010

[4] Kim H.Y., Nam S.Y., Hwang S.Y., Kim H.M. and Jeong H.J. (2016) Atractylone, an active constituent of KMP6, attenuates allergic inflammation on allergic rhinitis in vitro and in vivo models. Mol. Immunol. 78, 121–132 10.1016/j.molimm.2016.09.007 27636508

[5] Durham S.R. and Penagos M. (2016) Sublingual or subcutaneous immunotherapy for allergic rhinitis? J. Allergy Clin. Immunol. 137, 339–349.e10, 10.1016/j.jaci.2015.12.1298 26853126

[6] Tsai M., Grimbaldeston M. and Galli S.J. (2011) Mast cells and immunoregulation/immunomodulation. Adv. Exp. Med. Biol. 716, 186–211 10.1007/978-1-4419-9533-9_11 21713658

[7] Galli S.J. and Tsai M. (2012) IgE and mast cells in allergic disease. Nat. Med. 18, 693–704 10.1038/nm.2755 22561833 PMC3597223

[8] Amin K. (2012) The role of mast cells in allergic inflammation. Respir. Med. 106, 9–14 10.1016/j.rmed.2011.09.007 22112783

[9] Kobayashi N., Terada N., Hamano N., Numata T. and Konno A. (2000) Transepithelial migration of activated eosinophils induces a decrease of E-cadherin expression in cultured human nasal epithelial cells. Clin. Exp. Allergy 30, 807–817 10.1046/j.1365-2222.2000.00827.x 10848899

[10] Deng Y.Q., Yang Y.Q., Wang S.B., Li F., Liu M.Z., Hua Q.Q. (2015) Intranasal administration of lentiviral miR-135a regulates mast cell and allergen-induced inflammation by targeting GATA-3. PLoS ONE 10, e0139322 10.1371/journal.pone.0139322 26418311 PMC4587974

[11] Panganiban R.P., Wang Y., Howrylak J., Chinchilli V.M., Craig T.J., August A. (2016) Circulating microRNAs as biomarkers in patients with allergic rhinitis and asthma. J. Allergy Clin. Immunol. 137, 1423–1432 10.1016/j.jaci.2016.01.029 27025347

[12] Suojalehto H., Lindstrom I., Majuri M.L., Mitts C., Karjalainen J., Wolff H. (2014) Altered microRNA expression of nasal mucosa in long-term asthma and allergic rhinitis. Int. Arch. Allergy Immunol. 163, 168–178 10.1159/000358486 24513959

[13] Rebane A. (2015) microRNA and allergy. Adv. Exp. Med. Biol. 888, 331–352 10.1007/978-3-319-22671-2_17 26663191

[14] Xiao L., Jiang L., Hu Q. and Li Y. (2017) MicroRNA-133b ameliorates allergic inflammation and symptom in murine model of allergic rhinitis by targeting Nlrp3. Cell. Physiol. Biochem. 42, 901–912 10.1159/000478645 28662502

[15] Noh K., Kim M., Kim Y., Kim H., Kim H., Byun J. (2017) miR-122-SOCS1-JAK2 axis regulates allergic inflammation and allergic inflammation-promoted cellular interactions. Oncotarget 8, 63155–63176 10.18632/oncotarget.19149 28968979 PMC5609911

[16] Nishimura K., Ohtaka M., Takada H., Kurisaki A., Tran N.V.K., Tran Y. T.H. (2017) Simple and effective generation of transgene-free induced pluripotent stem cells using an auto-erasable Sendai virus vector responding to microRNA-302. Stem Cell Res. 23, 13–19 10.1016/j.scr.2017.06.011 28666145

[17] Parr C.J., Katayama S., Miki K., Kuang Y., Yoshida Y., Morizane A. (2016) MicroRNA-302 switch to identify and eliminate undifferentiated human pluripotent stem cells. Sci. Rep. 6, 32532 10.1038/srep32532 27608814 PMC5016789

[18] Zhang Z., Hong Y., Xiang D., Zhu P., Wu E., Li W. (2015) MicroRNA-302/367 cluster governs hESC self-renewal by dually regulating cell cycle and apoptosis pathways. Stem Cell Rep. 4, 645–657 10.1016/j.stemcr.2015.02.009 25801506 PMC4400607

[19] Anokye-Danso F., Trivedi C.M., Juhr D., Gupta M., Cui Z., Tian Y. (2011) Highly efficient miRNA-mediated reprogramming of mouse and human somatic cells to pluripotency. Cell Stem Cell 8, 376–388 10.1016/j.stem.2011.03.001 21474102 PMC3090650

[20] Nam S.Y., Kim M.H., Seo Y., Choi Y., Jang J.B., Kang I.C. (2014) The (2′S,7′S)-O-(2-methylbutanoyl)-columbianetin as a novel allergic rhinitis-control agent. Life Sci. 98, 103–112 10.1016/j.lfs.2014.01.003 24447626

[21] Kim M.H., Seo J.H., Kim H.M. and Jeong H.J. (2014) Zinc oxide nanoparticles, a novel candidate for the treatment of allergic inflammatory diseases. Eur. J. Pharmacol. 738, 31–39 10.1016/j.ejphar.2014.05.030 24877691

[22] Jung H.W., Jung J.K. and Park Y.K. (2011) Antiallergic effect of Ostericum koreanum root extract on ovalbumin-induced allergic rhinitis mouse model and mast cells. Asian Pac. J. Allergy Immunol. 29, 338–348 22299314

[23] Shakoory B., Fitzgerald S.M., Lee S.A., Chi D.S. and Krishnaswamy G. (2004) The role of human mast cell-derived cytokines in eosinophil biology. J. Interferon Cytokine Res. 24, 271–281 10.1089/107999004323065057 15153310

[24] Karin M. (1999) The beginning of the end: IkappaB kinase (IKK) and NF-kappaB activation. J. Biol. Chem. 274, 27339–27342 10.1074/jbc.274.39.27339 10488062

[25] Dissanayake E. and Inoue Y. (2016) MicroRNAs in allergic disease. Curr. Allergy Asthma Rep. 16, 67 10.1007/s11882-016-0648-z 27585977

[26] Lu T.X., Hartner J., Lim E.J., Fabry V., Mingler M.K., Cole E.T. (2011) MicroRNA-21 limits in vivo immune response-mediated activation of the IL-12/IFN-gamma pathway, Th1 polarization, and the severity of delayed-type hypersensitivity. J. Immunol. 187, 3362–3373 10.4049/jimmunol.1101235 21849676 PMC3175642

[27] Biethahn K., Orinska Z., Vigorito E., Goyeneche-Patino D.A., Mirghomizadeh F., Foger N. (2014) miRNA-155 controls mast cell activation by regulating the PI3Kgamma pathway and anaphylaxis in a mouse model. Allergy 69, 752–762 10.1111/all.12407 24734904

[28] Liu F., Qin H.B., Xu B., Zhou H. and Zhao D.Y. (2012) Profiling of miRNAs in pediatric asthma: upregulation of miRNA-221 and miRNA-485-3p. Mol. Med. Rep. 6, 1178–1182 10.3892/mmr.2012.1030 22895815

[29] Chiba Y., Tanabe M., Goto K., Sakai H. and Misawa M. (2009) Down-regulation of miR-133a contributes to up-regulation of Rhoa in bronchial smooth muscle cells. Am. J. Respir. Crit. Care Med. 180, 713–719 10.1164/rccm.200903-0325OC 19644046

[30] Tang H., Jiang H., Zheng J., Li J., Wei Y., Xu G. (2015) MicroRNA-106b regulates pro-allergic properties of dendritic cells and Th2 polarisation by targeting early growth response-2 in vitro. Int. Immunopharmacol. 28, 866–874 10.1016/j.intimp.2015.03.043 25864617

[31] Zhang X.H., Zhang Y.N., Li H.B., Hu C.Y., Wang N., Cao P.P. (2012) Overexpression of miR-125b, a novel regulator of innate immunity, in eosinophilic chronic rhinosinusitis with nasal polyps. Am. J. Respir. Crit. Care Med. 185, 140–151 10.1164/rccm.201103-0456OC 22071331

[32] Chen R.F., Huang H.C., Ou C.Y., Hsu T.Y., Chuang H., Chang J.C. (2010) MicroRNA-21 expression in neonatal blood associated with antenatal immunoglobulin E production and development of allergic rhinitis. Clin. Exp. Allergy 40, 1482–1490 10.1111/j.1365-2222.2010.03592.x 20701609

[33] Lu T.X., Lim E.J., Wen T., Plassard A.J., Hogan S.P., Martin L.J. (2012) MiR-375 is downregulated in epithelial cells after IL-13 stimulation and regulates an IL-13-induced epithelial transcriptome. Mucosal Immunol. 5, 388–396 10.1038/mi.2012.16 22453679 PMC4154234

[34] Mayoral R.J., Pipkin M.E., Pachkov M., van Nimwegen E., Rao A. and Monticelli S. (2009) MicroRNA-221-222 regulate the cell cycle in mast cells. J. Immunol. 182, 433–445 10.4049/jimmunol.182.1.433 19109175 PMC2610349

[35] Wang Q., Zhao D.Y., Xu H., Zhou H., Yang Q.Y., Liu F. (2015) Down-regulation of microRNA-223 promotes degranulation via the PI3K/Akt pathway by targeting IGF-1R in mast cells. PLoS ONE 10, e0123575 10.1371/journal.pone.0123575 25875646 PMC4395227

[36] Oh H.A., Kim H.M. and Jeong H.J. (2012) Alleviation of allergic rhinitis symptoms with Pyeongwee-San extract (KMP6). Immunopharmacol. Immunotoxicol. 34, 135–142 10.3109/08923973.2011.587128 21668288

[37] Heilker R., Freuler F., Pulfer R., Di Padova F. and Eder J. (1999) All three IkappaB isoforms and most Rel family members are stably associated with the IkappaB kinase 1/2 complex. Eur. J. Biochem. 259, 253–261 10.1046/j.1432-1327.1999.00028.x 9914500

[38] Yang Z., Zeng B., Wang C., Wang H., Huang P. and Pan Y. (2017) MicroRNA-124 alleviates chronic skin inflammation in atopic eczema via suppressing innate immune responses in keratinocytes. Cell. Immunol. 319, 53–60 10.1016/j.cellimm.2017.08.003 28847568

[39] Wu F. and Cui L. (2017) Resveratrol suppresses melanoma by inhibiting NF-kappaB/miR-221 and inducing TFG expression. Arch. Dermatol. Res. 309, 823–831 10.1007/s00403-017-1784-6 28936555

[40] Wang F., Jiang H., Wang S. and Chen B. (2017) Dual functional microRNA-186-5p targets both FGF2 and RelA to suppress tumorigenesis of glioblastoma multiforme. Cell. Mol. Neurobiol. 37, 1433–1442 10.1007/s10571-017-0474-4 28213656 PMC11482140

