# Expression of Concern: MiR-302e attenuates allergic inflammation in vitro model by targeting RelA

**DOI:** 10.1042/BSR-2018-0025_EOC

**Published:** 2024-04-12

**Keywords:** allergic inflammation, cytokines, mast cells, microRNA-302e, ReIA

The Editorial Office has been made aware of potential issues surrounding the scientific validity of this paper, hence has issued an expression of concern to notify readers whilst the Editorial Office investigates. It has been noted that Figure 4D GAPDH and Figure 4E RelA panels in this paper seem to appear as Figure 3A GAPDH and Figure 3A TIMP-3 panels in a paper by Shen et al 2018 (DOI: 10.3727/096504018X15201099883047).

