# Supplementary material for: MiR-302e attenuates allergic inflammation in vitro model by targeting RelA
Source: Biosci Rep. 2018 May 28;38(3):BSR20180025. doi: 10.1042/BSR20180025 (PMC6435536; doi:10.1042/BSR20180025)
Supplement: Supplementary file 1 [file bsr20180025_Supp1.pdf]

MiR-302e attenuates allergic inflammation in vitro model by targeting RelA

Lifeng Xiao[a], Li Jiang[a], Qi Hu[a], Yuru Li[a]

[a]*Department of Otolaryngology Head and Neck surgery, the First Affiliated Hospital of Harbin Medical University, Harbin, 150001, People's Republic of China*

Running title: MiR-302e inhibits allergic inflammation

Address correspondence to: Yuru Li, Department of Otolaryngology Head and Neck surgery, the First Affiliated Hospital of Harbin Medical University, NO. 23 Youzheng Street, Nangang District, Harbin, 150001, Heilongjiang Province, People's Republic of China. Tel: +86 0451-53643849. Fax: +86 0451-53643849. E-mail: liyr_hrbmu@163.com

Supplementary Materials

Figure S1.

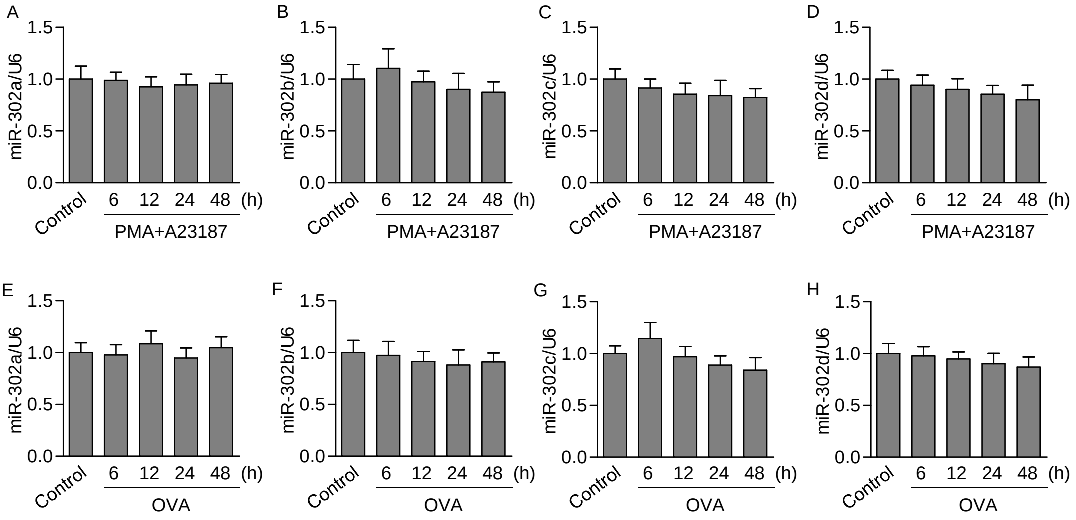

Figure S1. Effects of PMA/A23187 or OVA treatment on miR-302a, miR-302b, miR-302c and miR-302d level. (A-H) HMC-1 cells were treated with PMA (20 nM) plus A23187 (1 μM) or ovalbumin (OVA, 5 mg/mL) for 6, 12, 24 or 48 h. The expression of miR-302a (A and E), miR-302b (B and F), miR-302c (C and G) and miR-302d (D and H) was determined by quantitative real-time PCR.

Figure S2

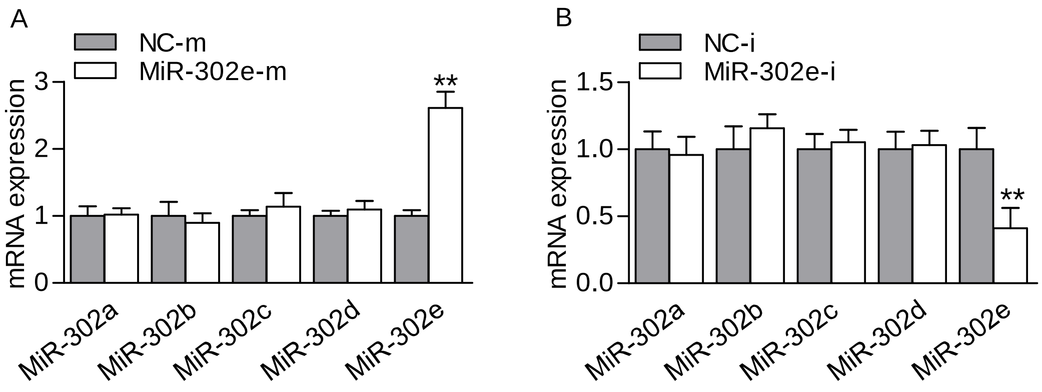

Figure S2. Effect of MiR-302e mimics or inhibitor on the expression of miR-302 family members. (A and B) HMC-1 cells transfected with miR-302 mimics (miR-302e-m, 40 nM) (A) or miR-302 inhibitor (miR-302e-i, 40 nM) (B) for 48 h. The expression of miR-302a, miR-302b, miR-302c, miR-302d and miR-302e was determined by quantitative real-time PCR. **P<0.01 vs. NC-m or NC-i, n=6.

Figure S3

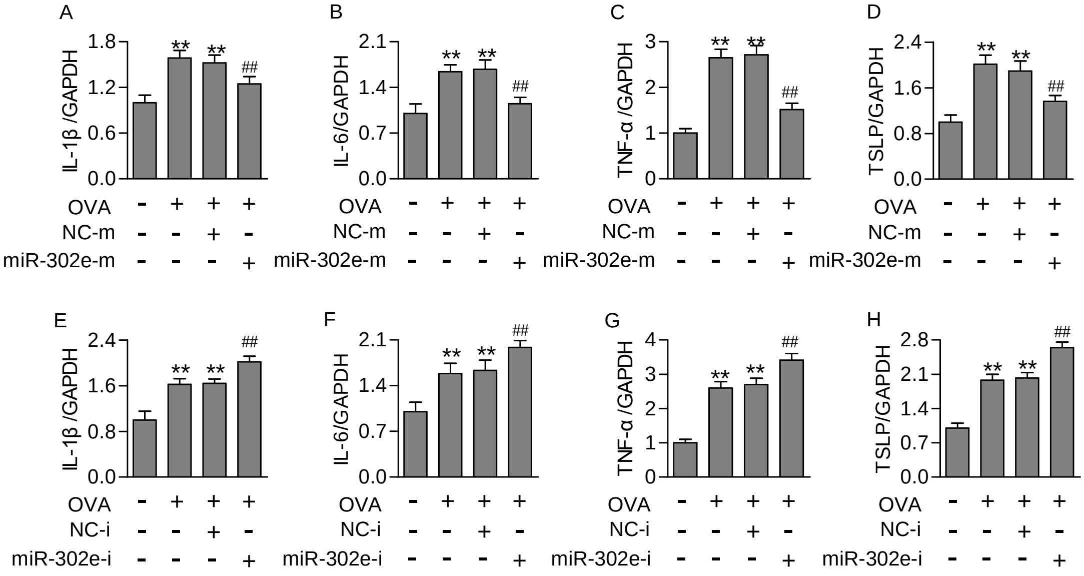

Figure S3. Effects of miR-302e on OVA-induced the mRNA level of cytokines. (A) HMC-1 cells were transfected with miR-302e mimics (miR-302e-m, 40 nM), miR-302e inhibitor (miR-302e-i, 40 nM), mimics negative control (NC-m) or inhibitor negative control (NC-i) for 48 h and then treated with ovalbumin (OVA, 5 mg/mL) for 24 h. The mRNA level of IL-1β (A and E), IL-6 (B and F), TNF-α (C and G) and TSLP (D and H) was examined by quantitative real-time PCR. **P<0.01 vs. control; ##P<0.01 vs. OVA, n=6.
